# Perineural Invasion As the Sole Pathologic Risk Factor After Surgical Resection for Head and Neck Squamous Cell Carcinoma

**DOI:** 10.7759/cureus.13094

**Published:** 2021-02-03

**Authors:** Ryan T Hughes, Joshua Farris, Cole Steber, Bart A Frizzell, Kathryn M Greven

**Affiliations:** 1 Radiation Oncology, Wake Forest School of Medicine, Winston-Salem, USA

**Keywords:** perineural spread, perineural invasion, head and neck neoplasms, squamous cell carcinoma

## Abstract

Introduction

Postoperative radiotherapy (PORT) is routinely recommended for patients with head and neck squamous cell carcinoma (HNSCC) based on pathologic risk factors (pRFs) such as perineural invasion (PNI). Patients with PNI as the sole pRF after resection of HNSCC are uncommon and their prognosis is less clear. The aim of this study is to assess the role of PNI as a sole risk factor in patients with otherwise pathologically low-risk HNSCC.

Methods

Patients with HNSCC of the oral cavity, pharynx, or larynx treated with primary surgical resection from 2013 to 2018 were identified from an institutional cancer registry. Those with pRFs (pathologic T3-4 disease, lymphovascular space invasion [LVSI], multiple positive lymph nodes, close [within 2 mm] or positive margins, extranodal extension [ENE], or recurrent disease) were excluded, yielding an otherwise pathologically low-risk cohort with or without incidental, pathologic PNI. Locoregional control (LRC), overall survival (OS) and disease-specific survival (DSS) were estimated and compared between PNI groups and by adjuvant therapy.

Results

A total of 1,058 patients were identified as having undergone surgical resection. Exclusion of patients with other pRFs, those with unknown PNI, and oral cavity patients with depth of invasion > 10 mm yielded a study cohort of 85 patients. Eight patients (10% of study group, <1% of all patients) had PNI as the sole pRF, none of which had clinical signs or symptoms of perineural tumor spread. The remaining 77 were negative for PNI and thus pathologically low risk. Patients with PNI were more likely to have oral cavity cancer, to be younger, and to have received PORT than those without PNI; no patient received concurrent chemotherapy. At a median follow-up of 46.4 months, two- and five-year LRC rates were 81.4% and 78.5%, respectively. No differences were noted between PNI-positive and PNI-negative groups (p=0.73) or PORT v. no-PORT groups (p=0.39). While the utility of PORT is not possible to assess given limited sample size, four patients with PNI who did not receive PORT did not experience locoregional failure. Seventeen patients overall experienced locoregional failure and 14 were ultimately salvaged. Five-year OS and DSS were 77.4% and 90.8%, respectively.

Conclusion

Patients with pathologically low-risk HNSCC after surgical resection experience high rates of LRC. In this large institutional cohort, PNI as the sole pRF was exceedingly rare, and the benefit of adjuvant therapies is difficult to assess. Within this limitation, PORT remains the standard of care for patients with PNI to reduce the risk of locoregional failure. Further collaborative studies are required to adequately assess the prognostic impact of PNI alone in resected HNSCC.

## Introduction

Perineural invasion (PNI), defined as tumor cell invasion into, around, or through nerves, has long been considered a pathologic risk factor (pRF) for recurrence after resection of head and neck squamous cell carcinoma (HNSCC) [1-7]. PNI is often identified in association with other high-risk pathologic features including lymphovascular space invasion (LVSI) and lymph node metastases and as a result has been considered a high-risk factor for local disease recurrence [3,7,8]. Early studies of postoperative radiotherapy (PORT) utilized risk stratification systems that included PNI to guide the use of PORT [9,10]. Favorable long-term outcomes after risk-adapted adjuvant radiotherapy, particularly for patients with low-risk pathologic features such as PNI alone [11]. Additionally, currently ongoing clinical trials utilize PNI as an indication for PORT (NCT00956007, NCT01898494, NCT02215265). Few studies have isolated the risk factor in the context of otherwise low-risk pathologic features to assess its individual impact on locoregional disease control. As a result, limited data exist regarding the prognostic role of PNI as the sole pRF [12]. In this study, we aimed to assess the frequency of PNI as the sole pRF as well as the prognostic impact of PNI on a cohort of otherwise pathologically low-risk patients after surgical resection for HNSCC.

## Materials and methods

In this Institutional Review Board-approved retrospective cohort study, all patients treated consecutively with surgical resection for HNSCC of the oral cavity, pharynx, or larynx between 2013 and 2018 were identified from an institutional cancer registry. Patients with pRFs other than PNI were excluded. These risk factors included pT3-4 (American Joint Committee on Cancer 7th Edition) disease, LVSI, close (within 2 mm) or positive margins, multiple positive lymph nodes, extranodal extension (ENE), or recurrent disease were excluded. Patients with oral cavity primary tumors with extensive depth of invasion (DOI) defined as greater than 10 mm were also excluded, as DOI is an independent risk factor in these patients [13]. Those with unknown or not reported PNI status after a review of the pathology report were excluded.

Locoregional recurrence was defined as clinical or pathological evidence of disease at the primary tumor site or neck. Locoregional control (LRC) was defined as the time from surgery to locoregional recurrence or last follow-up (right censor). Overall survival (OS) was defined as the duration from surgery to death from any cause or last follow-up. Disease-specific survival (DSS) was defined as the time from surgery to death due to the same disease.

Descriptive analyses were performed and compared between groups using the chi-square and Fisher’s exact test as appropriate for categorical variables and the Mann-Whitney U test for continuous variables. Time-to-event outcomes were estimated using the Kaplan-Meier method and compared across strata using the log-rank test. Statistical analyses were performed using R version 3.6 (R Foundation for Statistical Computing, Vienna, Austria).

## Results

A total of 1,058 patients were treated with upfront surgical therapy. Excluding patients with other high-risk pathologic features (pT3-4 disease, LVSI, close or positive margins, multiple lymph nodes or ENE) or recurrent disease yielded a group of 100 patients. Seven patients did not have PNI reported in the pathology report and were categorized as unknown and excluded, as were eight patients with high-risk oral cavity cancers with DOI > 10 mm. The remaining 85 patients (8.0% of all identified patients) formed the study cohort. Patient baseline characteristics are described in Table 1. Overall, eight patients (10.4% of the study cohort, 0.8% of all patients treated within this timeframe) had incidental PNI as the sole pRF; 77 patients were negative for PNI and thus pathologically low risk. There were no differences in gender, smoking status, HPV status, pathologic T or N stage between groups that did or did not have PNI as the sole pRF. Patients with PNI had oral cavity primary tumors (p=0.10) and were younger than patients without PNI (p<0.01). Nine of 13 (69.3%) oropharynx cancer patients with known HPV status had HPV-associated disease. Those with PNI were more likely to receive PORT: four of eight (50.0%) with PNI versus six of 77 (7.8%) without PNI (p<0.01). No patients received concurrent chemotherapy. Median follow-up was 46.4 months (95% CI 7.8-85.3).

**Table 1:** Patient Characteristics and Treatment by Perineural Invasion Gy: Gray; PNI: perineural invasion; NA: not applicable. All staging according to the American Joint Committee on Cancer 7th Edition.

		Overall	PNI Negative	PNI Positive	P-value
Total		85	77	8	
Age (median [range])		66.0 [20.0, 97.0]	66.0 [20.0, 97.0]	53.0 [36.0, 68.0]	<0.01
Sex (%)	Female	32 (37.6)	29 (37.7)	3 (37.5)	1.00
	Male	53 (62.4)	48 (62.3)	5 (62.5)	
Site Category (%)	Oral Cavity	53 (62.4)	45 (58.4)	8 (100.0)	0.10
	Oropharynx	17 (20.0)	17 (22.1)	0 (0.0)	
	Larynx/Hypopharynx	15 (17.6)	15 (19.5)	0 (0.0)	
Tumor Site (%)	Base of Tongue	6 (7.1)	6 (7.9)	0 (0.0)	0.05
	Buccal Mucosa	5 (6.0)	5 (6.6)	0 (0.0)	
	Floor of Mouth	5 (6.0)	4 (5.3)	1 (12.5)	
	Gingiva	7 (8.3)	6 (7.9)	1 (12.5)	
	Glottic Larynx	6 (7.1)	6 (7.9)	0 (0.0)	
	Lip	5 (6.0)	3 (3.9)	2 (25.0)	
	Posterior Pharyngeal Wall	1 (1.2)	1 (1.3)	0 (0.0)	
	Retromolar Trigone	1 (1.2)	0 (0.0)	1 (12.5)	
	Supraglottic Larynx	7 (8.3)	7 (9.2)	0 (0.0)	
	Soft Palate	1 (1.2)	1 (1.3)	0 (0.0)	
	Tongue	30 (35.7)	27 (35.5)	3 (37.5)	
	Tonsil	10 (11.9)	10 (13.2)	0 (0.0)	
Tobacco Use (%)	Current/Former	56 (65.9)	48 (62.3)	8 (100.0)	0.10
	Never	23 (27.1)	23 (29.9)	0 (0.0)	
	Smokeless Only	6 (7.1)	6 (7.8)	0 (0.0)	
Pathologic T Stage (%)	pT1	49 (57.6)	44 (57.1)	5 (62.5)	1.00
	pT2	36 (42.4)	33 (42.9)	3 (37.5)	
Pathologic N Stage (%)	pN0	69 (81.2)	63 (81.8)	6 (75.0)	0.64
	pN1	16 (18.8)	14 (18.2)	2 (25.0)	
Neck Dissection (%)	No	10 (11.8)	10 (13.0)	0 (0.0)	0.61
	Yes	75 (88.2)	67 (87.0)	8 (100.0)	
Radiotherapy (%)	No	75 (88.2)	71 (92.2)	4 (50.0)	<0.01
	Yes	10 (11.8)	6 (7.8)	4 (50.0)	
Radiotherapy Neck Target (%)	Bilateral Neck	5 (50.0)	3 (50.0)	2 (50.0)	0.33
	Primary Only	3 (30.0)	1 (16.7)	2 (50.0)	
	Unknown	2 (20.0)	2 (33.3)	0 (0.0)	
Radiotherapy Dose (Gy) (median [range])		60.0 [60.0, 60.0]	60.0 [60.0, 60.0]	60.0 [60.0, 60.0]	NA

**Figure 1:**
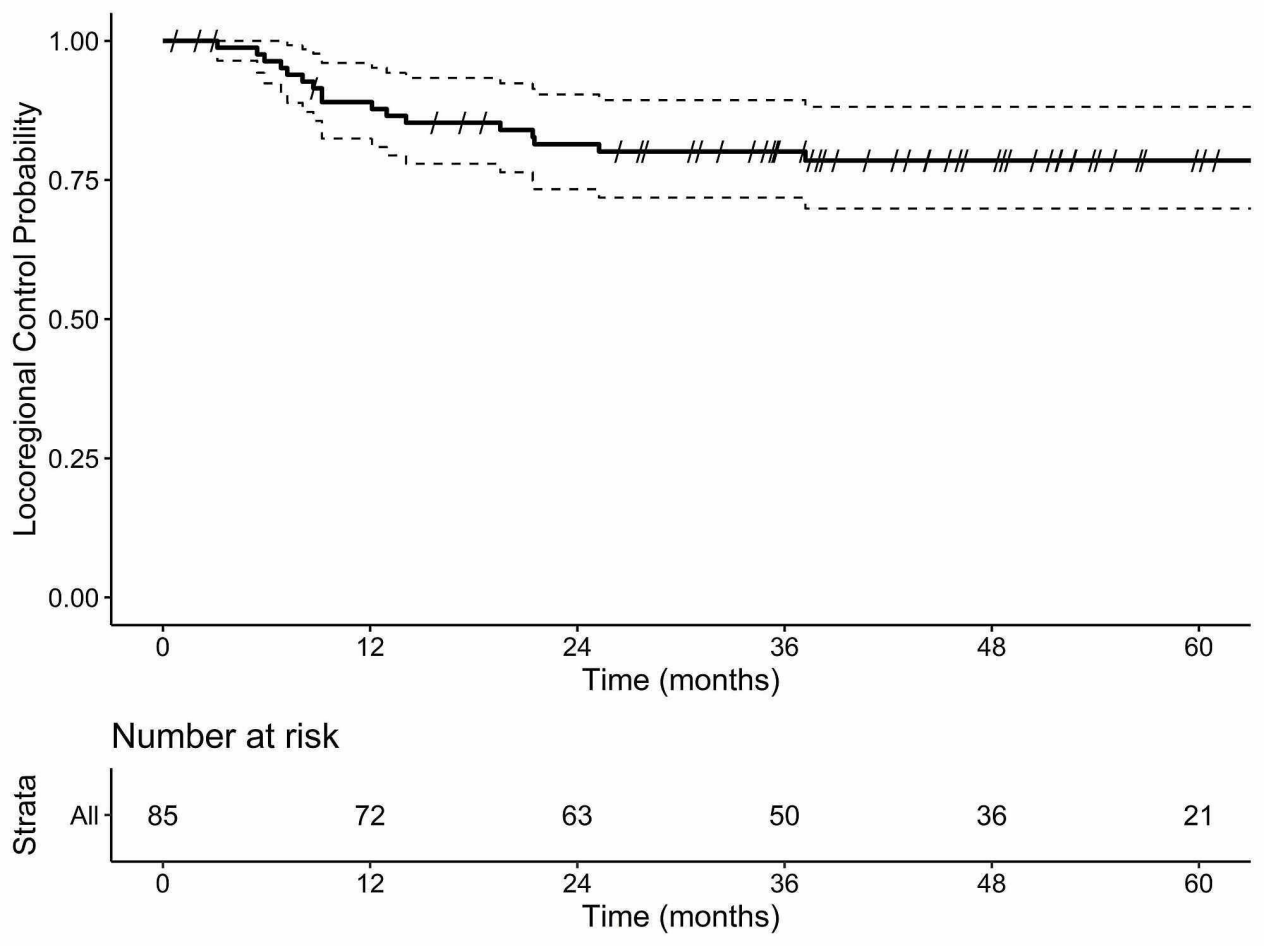
Kaplan-Meier plot of locoregional control after primary surgical management of head and neck squamous cell cancer in pathologically low-risk patients irrespective of PNI status. PNI: perineural invasion

In total, 17 patients experienced locoregional recurrence at a median time to recurrence of 9.2 months (95% CI 5.0-27.7). Patterns of locoregional recurrence were as follows: local only (n=6), regional only (n=6), and local and regional (n=5). Kaplan-Meier estimates of LRC at two and five years were 81.4% (95% CI 73.3-90.4) and 78.5% (95% CI 69.9-88.2), respectively (Figure 1). There were no differences in LRC between PNI-positive and PNI-negative groups (p=0.73). LRC was not impacted by PORT (p=0.39). Of the four patients with PNI as the sole pRF that did not receive PORT, none experienced locoregional failure. Due to the low number of PNI positive patients, comparisons within that group were not possible. Of the 17 patients that failed, 14 were salvaged with surgical resection alone (n=8), surgery followed by PORT (n=2), resection followed by PORT with concurrent chemotherapy (n=2), or RT alone (n=2).

**Figure 2:**
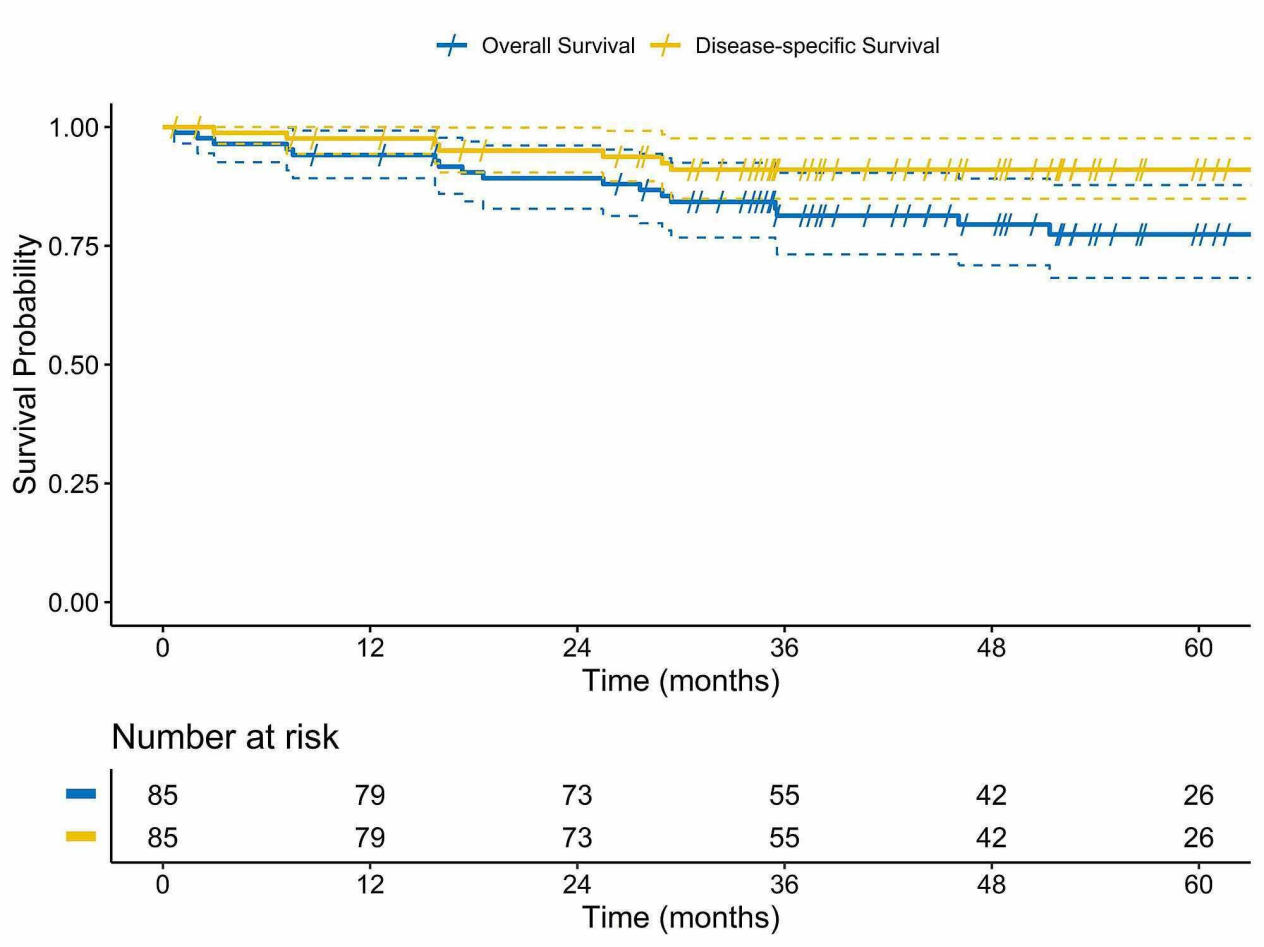
Kaplan-Meier plot of overall survival and disease-specific survival.

Two- and five-year OS rates were 89.2% (95% CI 82.8-96.1) and 77.4% (95% CI 68.2-87.8), respectively. Corresponding estimates of DSS were 95.1% (95% CI 90.4-99.9) and 90.8% (95% CI 84.9-97.6), respectively (Figure 2).

## Discussion

Radiation therapy is routinely recommended for resected HNSCC with pRFs including pT3-4 disease, multiple lymph nodes, LVSI, PNI, close/positive margins, and ENE [14,15]. These risk factors frequently occur in combination and portend an increased risk of locoregional failure after surgery alone. In rare cases (less than 1% of patients treated at our institution), the tumor is positive for PNI as the sole pRF in the absence of other high-risk features. In these cases, the optimal postoperative approach is difficult to determine. Not surprisingly, PORT was given to half the patients with PNI as the sole risk factor. Notably, the LRC identified in our study is consistent with prior reports of low-risk patients [4,9,10]. However, the fact that four of eight patients did not receive PORT demonstrates the relevance of this issue.

A study evaluating the prognostic impact of PNI on patients with surgically resected oral cavity cancers with pathologically negative necks found that PNI was associated with worse LRC and disease-free interval [5]. However, the multivariable analyses identifying these associations included high grade and perivascular invasion, the frequency of which as they relate to PNI were not reported. Due to its frequently strong association with other pRFs, analyses including unselected patients with a variety of risk factors are limited in their ability to truly discriminate the impact of PNI itself [8,16]. Further large, multi-institutional studies are needed to assess the impact of PNI as the sole risk factor on patients with resected HNSCC.

A factor worth consideration is the extent and size of PNI in the tumor specimen. Large-nerve involvement and extensive PNI are frequently associated with disease recurrence in cutaneous HNSCC [17-19]. A pathologic risk assessment tool for oral cavity squamous cell carcinoma that included the size of nerves involved (in addition to lymphocytic tumoral infiltrate and worst pattern of invasion) has been associated with survival and disease-free survival [4,20]. Large nerve involvement was categorized as carcinoma tracking along or within a nerve with a diameter ≥ 1 mm, while small nerve involvement included only nerves < 1 mm in diameter. The definition of extensive PNI is inconsistent in the literature but often is applied in the case of involvement of ≥2 nerves [21]. Given the lack of clear evidence in the literature, adjuvant radiotherapy should remain a strong consideration, particularly in the presence of large nerve or multifocal PNI [12]. These considerations do not take into account clinical or radiographic perineural tumor spread from mucosal, salivary, or cutaneous malignancies, all of which warrant PORT [21]. This study specifically evaluates the impact of incidental pathologic PNI (without clinical findings suggestive of nerve involvement) on disease control.

This study is limited by its retrospective nature and the small sample size of pathologically low-risk patients with or without PNI. By limiting the analysis to pathologically low-risk patients with or without PNI and excluding other commonly accepted risk factors, we attempted to minimize any confounding factors and isolate PNI as the single pRF of interest. This limited our power to potentially identify differences between groups with regard to PNI and the use of PORT and introduces potential selection bias with regard to the utility of adjuvant therapy. Our institution’s large overall head and neck patient volume allowed us to identify and describe a rare circumstance occurring in <1% of patients undergoing upfront surgical resection.

## Conclusions

PNI as the sole pRF after resection of HNSCC is an extremely rare circumstance. It is associated with younger age, oral cavity primary tumor, and the receipt of PORT. Since very few patients with PNI did not receive PORT, no conclusions can be made regarding its utility in this scenario, and it remains the standard of care. Further study is warranted, optimally including multiple institutions to pool data and improve statistical power.
